# Case of Abdominal Inflammation, Principally in the Kidneys and Bladder, Occasioned by Excessive Debauchery

**Published:** 1814-05

**Authors:** William Jones

**Affiliations:** London


*For the Medical and Physical Journal.*

### *Case of Abdominal Inflammation, principally in the Kidneys and Bladder, occasioned by excessive Debauchery.*

MR. A. B., after violent and continued debauches, was taken ill on the 25th of the month. He was hot, with a sense of lassitude and torpor in his limbs, and exquisite sensibility of the surface of the belly, but principally on the pit of the stomach; he complained also of acute pains in the right kidney, with inability to pass his urine; his bowels were costive. Warm baths, emollient enemas, were administered, and a bladder full of cold water was applied to the stomach. Six or eight hours after, a vomiting of bilious matter took place, which produced great debility, and his night was passed without sleep. The symptoms continued in this state till the 30th, (upwards of five days.) He was now yellow; his eyes were sunk and hollow; he had a bitter taste in his mouth; the tongue was loaded with a yellow bilious matter, a considerable quantity of which was vomited all night. The pains in the loins were most acute, with obstinate constipation, and total retention of urine. The pulse was frequent, small, and hard; in addition to these, he was distressed by spasmodic contractions of the muscles, and a general sensibility of the abdomen was so great, that pressure made for the ordinary purpose of examination could not be borne.

The violence of the symptoms left us no hope of recovery. Emollient drinks and enemas were prescribed, with cordial and antispasmodic draughts. During the morning he expectorated a quantity of bilious matter, which he had before vomited. At eleven o'clock the pains increased, at twelve were most excruciating, at one he became delirious, and at three he died.

On dissection several ecchymoses were discovered at the inferior surface of the heart, the cavities of which were emptied. The peritoneum appeared acutely inflamed, and covered with coagulable lymph. The abdominal viscera, except the liver, kidneys, and spleen, formed one entire mass; at some parts in a state of suppuration, but the greater part was glued together by coagulable lymph: neither the suppuration or inflammation extended beyond the peritoneal covering. The liver was sound, and the gall-bladder was filled with black bile. The spleen was healthy. The alimentary tube contained a yellow bilious fluid. The fat surrounding the kidneys was very vascular. The right kidney was healthy, but the left very red in its internal part. The pelvis had a greenish tint, and the commencement of the ureter was inflamed. The bladder contained a red and thick liquid, and was very much distended; its walls had acquired a considerable thickness; all its internal surface was entirely disorganised, and was lined with broad red and white flakes. The prostate gland was swoln. The urethra was not inflamed, excepting at those parts in the vicinity of the fossa navicularis.

WILLIAM JONES.

*London, April* 4, 1814.

